# Nasal septum-derived chondroprogenitor cells control mandibular condylar resorption consequent to orthognathic surgery: a clinical trial

**DOI:** 10.1093/stcltm/szae026

**Published:** 2024-04-12

**Authors:** Ricardo de Souza Tesch, Esther Rieko Takamori, Karla Menezes, Rosana Bizon Vieira Carias, Carmen Lucia Kuniyoshi Rebelatto, Alexandra Cristina Senegaglia, Debora Regina Daga, Leticia Fracaro, Anny Waloski Robert, Carlos Bruno Reis Pinheiro, Marcelo de Freitas Aguiar, Pablo Javier Blanco, Eduardo Guerreiro Zilves, Paulo Roberto Slud Brofman, Radovan Borojevic

**Affiliations:** Regenerative Medicine Laboratory, Petrópolis Medical School/ UNIFASE, Avenida Barão do Rio Branco 1003, Centro, Petrópolis, RJ 25680-120, Brazil; Regenerative Medicine Laboratory, Petrópolis Medical School/ UNIFASE, Avenida Barão do Rio Branco 1003, Centro, Petrópolis, RJ 25680-120, Brazil; Institute of Biomedical Sciences, Federal University of Rio de Janeiro, Ave. Carlos Chagas Filho 373, Sala B1-011, Cidade Universitária, Ilha Do Fundão, Rio de Janeiro, RJ 21941-590, Brazil; Regenerative Medicine Laboratory, Petrópolis Medical School/ UNIFASE, Avenida Barão do Rio Branco 1003, Centro, Petrópolis, RJ 25680-120, Brazil; Core for Cell Technology, School of Medicine and Life Sciences, Pontifícia Universidade Católica do Paraná, Prado Velho, Curitiba, PR 80215-901, Brazil; Core for Cell Technology, School of Medicine and Life Sciences, Pontifícia Universidade Católica do Paraná, Prado Velho, Curitiba, PR 80215-901, Brazil; Core for Cell Technology, School of Medicine and Life Sciences, Pontifícia Universidade Católica do Paraná, Prado Velho, Curitiba, PR 80215-901, Brazil; Core for Cell Technology, School of Medicine and Life Sciences, Pontifícia Universidade Católica do Paraná, Prado Velho, Curitiba, PR 80215-901, Brazil; Stem Cells Basic Biology Laboratory, Carlos Chagas Institute – FIOCRUZ/PR, Rua Professor Algacyr Munhoz Mader, 3775, Curitiba, PR, 81350-010, Brazil; Postgraduate Program in Exercise and Sport Sciences, State University of Rio de Janeiro, Rua São Francisco Xavier 524, Maracanã, Rio de Janeiro, RJ, 20550-013, Brazil; Department of Specific Formation, School of Dentistry, Health Institute of Nova Friburgo, Fluminense Federal University, Rua Dr. Silvio Henrique Braune, 22, Nova Friburgo, RJ, 28625-650, Brazil; Department of Mathematical and Computational Models, National Laboratory for Scientific Computing, Avenida Getúlio Vargas, 333, Petrópolis, RJ, 25651-075, Brazil; Department of Mathematical and Computational Models, National Laboratory for Scientific Computing, Avenida Getúlio Vargas, 333, Petrópolis, RJ, 25651-075, Brazil; Core for Cell Technology, School of Medicine and Life Sciences, Pontifícia Universidade Católica do Paraná, Prado Velho, Curitiba, PR 80215-901, Brazil; Regenerative Medicine Laboratory, Petrópolis Medical School/ UNIFASE, Avenida Barão do Rio Branco 1003, Centro, Petrópolis, RJ 25680-120, Brazil

**Keywords:** cellular therapy, progenitor cells, skeleton, clinical translational

## Abstract

Condylar resorption is an aggressive and disability form of temporomandibular joint (TMJ) degenerative disease, usually non-respondent to conservative or minimally invasive therapies and often leading to surgical intervention and prostheses implantation. This condition is also one of the most dreaded postoperative complications of orthognathic surgery, with severe cartilage erosion and loss of subchondral bone volume and mineral density, associated with a painful or not inflammatory processes. Because regenerative medicine has emerged as an alternative for orthopedic cases with advanced degenerative joint disease, we conducted a phase I/IIa clinical trial (U1111-1194-6997) to evaluate the safety and efficacy of autologous nasal septal chondroprogenitor cells. Ten participants underwent biopsy of the nasal septum cartilage during their orthognathic surgery. The harvested cells were cultured in vitro and analyzed for viability, presence of phenotype markers for mesenchymal stem and/or chondroprogenitor cells, and the potential to differentiate into chondrocytes, adipocytes, and osteoblasts. After the intra-articular injection of the cell therapy, clinical follow-up was performed using the Diagnostic Criteria for Temporomandibular Disorders (DC/TMD) and computed tomography (CT) images. No serious adverse events related to the cell therapy injection were observed during the 12-month follow-up period. It was found that autologous chondroprogenitors reduced arthralgia, promoted stabilization of mandibular function and condylar volume, and regeneration of condylar tissues. This study demonstrates that chondroprogenitor cells from the nasal septum may be a promise strategy for the treatment of temporomandibular degenerative joint disease that do not respond to other conservative therapies.

## Graphical Abstract

**Graphical Abstract:**
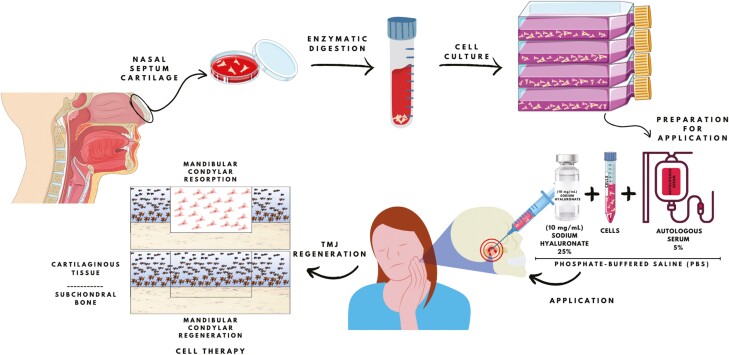

**Lessons Learned**

- A single intra-articular injection of autologous nasal septum cartilage progenitor cells was safe and well tolerated for the treatment of mandibular condylar resorption.
- This procedure was effective in controlling joint pain and mandibular functional disability.
- It was also possible to observe regenerative effects in damaged joint tissues.
- Therefore, this intervention can be considered a potential treatment for temporomandibular joint osteoarthritis refractory to other therapeutic options.

**Significance Statement**

The present study suggests that temporomandibular joint lavage followed by a single intra-articular injection of cartilage progenitor cells, harvested from the patient’s nasal septum, and suspended in a sodium hyaluronate vehicle, has the potential to be a safe and well-tolerated treatment for condylar resorption after orthognathic surgery. The results demonstrate improvement in joint pain and mandibular function, as well as favorable structural effects on the tissues of the temporomandibular joint affected by degenerative disease. This intervention is a potential modifier of temporomandibular joint osteoarthritis disease refractory to other therapeutic modalities.

## Introduction

Degenerative joint diseases (DJD) begin as cartilage erosions or loss of subchondral bone mineral density, associated with inflammatory processes, which may be its trigger or immunologic consequence.^1^ This process can be characterized as osteoarthritis, a progressive disease of the entire joint organ,^2^ with significant morbidity worldwide. Osteoarthritis may attack different synovial joints, including the temporomandibular joint (TMJ). The major clinical diagnostic criterion for TMJ intra-articular inflammation is the painful limitation of mouth opening.^3^ However, cases of subclinical osteoarthritis with a degenerative joint disease but without arthralgia can also occur, and their monitoring should be done by the follow-up of images.

TMJ computed tomography (CT) showed that cortical erosions and subchondral cysts are the main characteristics that differentiate patients from asymptomatic controls.^4^ A longitudinal study with a 5-year follow-up demonstrated that subchondral cysts tend to form in areas under osteoarthritis mechanical overload, mostly leading to bone resorption and deformation of the mandibular condyle.^5^ Studies of the natural course of severe TMJ osteoarthritis also showed that most discontinuous cortex erosions are converted to continuous ones for up to 2 years. However, there is also an increased probability of condylar volume reduction by resorption,^6^ even when they are conservatively treated^7^

The concept of condylar resorption (CR) has now gained attention. Whether this is a separate disease or a subtype of TMJ osteoarthritis has yet to be determined. The evidence of different etiologies of condylar resorption is relatively low, and its clinical features are comparable to those of severe degenerative joint diseases.^6^ This resorption is one of the significant post-surgical complications of orthognathic surgery, and the resorption degree of condylar height is related to post-operative skeletal relapse.^8^ Risk factors for condylar resorption following orthognathic surgery include disc displacements and degenerative lesions before treatment^9^ especially when mandibular advancement associated with counterclockwise rotation is done. The incidence of condylar resorption following mandibular osteotomies varies between studies, reaching up to 31%.^10^

Different protocols have been proposed to address the effects of condylar resorption after orthognathic surgery. They range from pharmacological therapies^11^ to surgical procedures involving the repositioning of the articular disc^12^ or even the total TMJ prostheses replacement.^13^ Regenerative medicine has proven to be an alternative for orthopedic cases involving advanced degenerative joint disease, and the knee has served as a translational research model for developing innovative TMJ therapies.^14^

Due to its avascular nature,^15^ the fibrocartilage covering the mandibular condyle has a limited capacity for self-repair. Mesenchymal progenitor cells reside in the outermost condyle layer and constitute the only cell reservoir able to participate in cartilage self-regeneration. During such processes, fibrocartilage chondroprogenitor stem cells’ proliferation, differentiation, and maturation are regulated by complex signals involving the Wnt/β-catenin pathway.^16^ However, the maintenance of non-physiological joint overload can lead to abnormal activity of this pathway and induce unexpected chondrocyte hypertrophy, leading to cartilage degeneration through increased expression of multiple catabolic factors.^17^

The use of the nasal septum as a source of cartilage mesenchymal stem cells has been previously described.^18^ In a first-in-human trial, these cells were harvested, matrix engineered, and applied to repair knee cartilage defects, efficiently integrating the grafted cells into the adjacent native cartilage and the underlying subchondral bone.^19^ Our group has proposed a novel treatment for condylar resorption after orthognathic surgery using autologous nasal septum stem cells transplantation and reported the first human case.^20^ In the present study, we describe the results of a phase I/IIA clinical trial evaluating the safety and efficacy of autologous chondroprogenitor cells derived from the nasal septum for the treatment of patients with TMJ condylar resorption associated with orthognathic surgery.

## Materials and methods

### Patients

The present study has been approved by the Petrópolis Medical School/ UNIFASE Committee of Ethics in Research (CEP-FMP/UNIFASE) and by the National Commission of Ethics in Medical Research (CONEP), CAAE 12484813.0.0000.5245. The study was retrospectively registered in the Brazilian National Clinical Trials Registry and the US Clinical Trials Registry under the Universal Trial Number (UTN) U1111-1194-6997. Inclusion criteria: Patients aged 18 or over, diagnosed with dentofacial deformities related to severe degenerative disease of the TMJ, irrespective of the presence or absence of concomitant arthralgia, and with a surgical indication for orthognathic surgery to correct the dentofacial deformity. Exclusion criteria: pregnant or breastfeeding patients, patients with local infection or other comorbidities that contraindicate the surgical procedure, rheumatic diseases, and chronic pain in a joint other than the temporomandibular

Patients were enrolled after signing an informed consent form. After indicating the regenerative medicine approach and enrollment in the present study, 10 research participants underwent a nasal cartilage biopsy to isolate and process chondrocytes derived from the nasal septum. This procedure was performed during the orthognathic surgery, under general anesthesia, and 20 mL of blood was collected from the patient to prepare autologous serum. All steps of this study followed a systematic timeline (Supplementary Figure S1).

The present open-label phase I/IIA clinical study aimed to evaluate the safety and efficacy of autologous chondroprogenitor cells derived from the nasal septum for treating patients with TMJ condylar resorption. This study is the first globally to administer these cells into the temporomandibular joint, its design involved a limited number of patients, and the gradual inclusion of participants was recommended by the National Research Ethics Commission (CONEP). The inclusion followed a careful scheme, starting with 2 patients, and the absence of complications within 3 months allowed the progressive inclusion of additional patients. After observing the absence of serious adverse events, we expanded the study to a total of 10 patients.

Ten participants were included, 9 received intra-articular injection of autologous chondroprogenitor cells, in both TMJs, while 1 participant (ID 8) could not be treated due to an in vitro cell contamination. Participants were recruited between January of 2016 and October of 2021. The average age of participants was 30.6 years; 7 females and 3 males. Participants were followed up for 12 months, being submitted to clinical evaluation using the DC/TMD for TMJ signs and symptoms.

### Nasal septum cartilage processing and quality control

Nasal cartilage biopsy processing, cell culture conditions, cell suspension for therapy, and cell transport conditions were prepared as described previously.^20^ All quality controls were performed on the final product before infusion. Sterility was evaluated by tests to detect bacteria and fungi (Bact/Alert 3D, Biomerieux), endotoxins (Endosafe PTS, Charles River), and mycoplasma (KIT MycoAlert PLUS for Mycoplasma Detection, Lonza). Cell viability controls were done by flow cytometry using the vital dye 7-aminoactinomycin D (7-AAD; BD #559925), to determine the percentage of viable cells, and Annexin V protein (BD #51-65,875X) to determine the percentage of cells in apoptosis. Cytogenetic analysis was done by the GTG-banding.

### Characterization of the nasal septum cartilage cell population

The cells were characterized before the clinical application to assess surface markers by flow cytometry. Cells were washed with PBS containing 2% FBS and incubated with the monoclonal antibodies: fluorescein isothiocyanate (FITC)-labeled CD14 (BD #555397), CD45 (BD #555482), CD19 (BD #555412), CD44 (BD #555478); phycoerythrin (PE)-labeled CD73 (BD #550257), CD90 (BD #555596), CD166 (BD #559263), CD151 (BD #556057), CD49C (BD #556025); PerCP-labeled HLADR (BD #551375); and APC-labeled CD34 (BD #555824), CD105 (BD #562408), CD29 (BD #559883), all purchased from BD (Pharmingen). At least 100 000 events were acquired on a BD FACSCalibur flow cytometer (BD Biosciences) and the data were analyzed using FlowJo 10 (TreeStar) software.

To evaluate the potential for cell differentiation into adipocytes and osteoblasts, cells were plated in triplicates (40 000 cells/well) in 24-well plates on glass coverslips to monitor their potential adipogenic and osteogenic differentiation, according to.^21^ Their potential of chondrogenic differentiation was assessed by micromass culture. Approximately 1.5 × 10^6^ cells in 1 mL of culture medium were centrifuged at 300*g* for 10 minutes in a conical tube to form a cell pellet. The collagen type II deposition areas were quantified using Image Pro-Plus 4.5 software (Media Cybernetics, Rockville, MD, USA), and the areas were converted to percentages.

### RNA extraction, cDNA synthesis, and quantitative PCR

After the chondrogenic differentiation, the cell aggregates were resuspended in TRI Reagent (Sigma) followed by addition of chloroform. After centrifugation, the aqueous phase was isolated, mixed with 70% ethanol and added to the purification columns from RNA isolation with PureLink RNA mini kit (Thermo Fisher), and the manufacturer’s instructions were followed. For each sample, 132 ng of RNA was used for the cDNA synthesis using the ImProm-II Reverse Transcription System (Promega), again according to the manufacturer’s instructions. cDNA amplification was done with GoTaq qPCR Master Mix (Promega) and the specific primers listed in Supplementary Table S1, in a final volume of 10 µL. Quantitative PCR (qPCR) was done in the QuantStudio 5 Real-Time PCR system (Thermo Fisher) and the results were analyzed with the QuantStudio Design and Analysis Software version 2.6.0 (Thermo Fisher). The Cq results for each gene were normalized based on RNA polymerase II (POLR2) expression.

The results were plotted as a relative expression using the 2 −ΔΔCt (fold change), comparing induced and non-induced cells and as the expression ratio to RNA polymerase II (POLR2) expression (fold to POLR2).

### Clinical injection of cells

Cells from passage 3 to 5 were suspended in phosphate-buffered saline (PBS), supplemented with 25% (v/v) of injectable solution containing 10 mg/mL sodium hyaluronate (Osteonil mini, TRB Pharma) and 5% autologous serum. The final volume injected in each TMJ was 1 mL containing 10^7^ cells after arthrocentesis, according to procedures described by De Souza Tesch et al.^20^

### Clinical follow-up

The same examiner carried out the clinical evaluation of the patients of this study at the time of diagnosis (initial clinical evaluation), and during the clinical follow-up period at 7 and 15 days, 1, 3, 6, and 12 months after application of the treatment, using “Diagnostic Criteria for Temporomandibular Disorders” (DC/TMD) for TMJ signs and symptoms.

### Images acquisition and condylar remodeling analysis

The images were acquired in a Cone Beam CT “I-Cat Classic” (Imaging Science International ) using the extended height protocol: field of view 16 × 22 cm, scan time 40 s, and voxel size 0.4 mm. The acquisition protocol was the same at T1 and T2 (obtained before and up 15 months post therapy, respectively) to avoid differences in image resolution. The acquired images were saved in DICOM file format. The ITK-SNAP 3.8.0 software (www.itksnap.org)^22^ was applied to the segmentation step, and 3D slicer 5.0.2 software (www.3dslicer.com)^23^ to register the scans and their respective 3 dimensional volumetric models, following the protocol used by De Souza Tesch et al^20^.

The visual analysis of 3-dimensional morphological and volumetric changes was done, using 3D Slicer 5.0.2 software with the extensions modules ModelToModelDistance and ShapePopulationViewer, which allow the measurement of the distance between two 3-dimensional models and to visualize and compare the surfaces at the same time.

These modules illustrate the difference between distances of 3-dimensional models (T1 e T2) through color variation, considering that green areas show some degree of resorption between the initial and final models, a yellow color identifies moderate changes, and a red color identifies marked differences between models indicating bone apposition.

After co-registration, both baseline and follow-up geometries were sliced within the same plane, removing any discrepancy in the original slicing. Then, for each geometry, the volume was computed based on the face normal values, using Blender 3.5 software, a 3D modelling and rendering package, (http://www.blender.org). The method was proposed by Zhang and Chen^24^ and allows the computation of the volume of a triangulated mesh using solely the triangle vertices.

## Results

The nasoseptal chondroprogenitor cells were positive for CD105, CD73, and CD90 surface markers, described for mesenchymal progenitor cells of different origins^25^ (Table 1). The CD44 hyaluronan receptor that organizes the pericellular matrix was also highly expressed in the majority of nasoseptal chondrogenic progenitor cells from all the research participants. Unexpectedly, the nasoseptal chondrogenic progenitors were positive in vitro for CD146 (passages 3 to 5) (Table 1), a perivascular cell marker first described in the bone marrow.^26^ We observed CD146 + in over 90% of the expanded nasal septum cells in the later passages (3 to 5), which were injected into the research participants. As an exception, only participant ID 9, 61 years old, had only half of the analyzed cell population positive for CD146 (50.8%) (Table 1). At the same time, the cluster of differentiation markers that usually characterize stem cells of the hematopoietic origin (CD14, CD19, CD34, and CD45) was nearly absent (Table 1).

**Table 1.** Percentage (%) of nasoseptal chondroprogenitor cells markers.

ID	CD105	CD73	CD90	CD44	CD146	CD14	CD19	CD34	CD45	CD49	CD151
01	65.1	87.2	99.8	82.5	97.6	0.9	0.6	0.3	0.4	95.1	96.8
02	99.3	99.3	100	99.6	99.0	0.4	0.5	0.6	0.9	99.9	99.9
03	99.0	98.6	100	99.1	89.6	0.1	0.2	0.1	0.2	99.0	99.8
04	98.0	99.7	99.9	99.9	91.2	0.2	0.2	0.3	0.1	100	100
05	99.9	100	99.8	99.9	95.1	0.0	0.1	1.6	0.7	99.9	100
06	99.3	99.8	100	99.8	93.1	0.2	0.6	0.9	1.6	100	100
07	99.9	99.9	100	99.6	94.9	0.0	0.4	0.3	0.0	99.8	99.8
09	94.4	98.9	99.4	99.8	50.8	0.2	0.0	0.2	0.1	99.6	99.3
10	98.9	99.9	100	99.8	90.9	0.0	0.6	0.6	0.9	100	99.9

**Figure 1.**
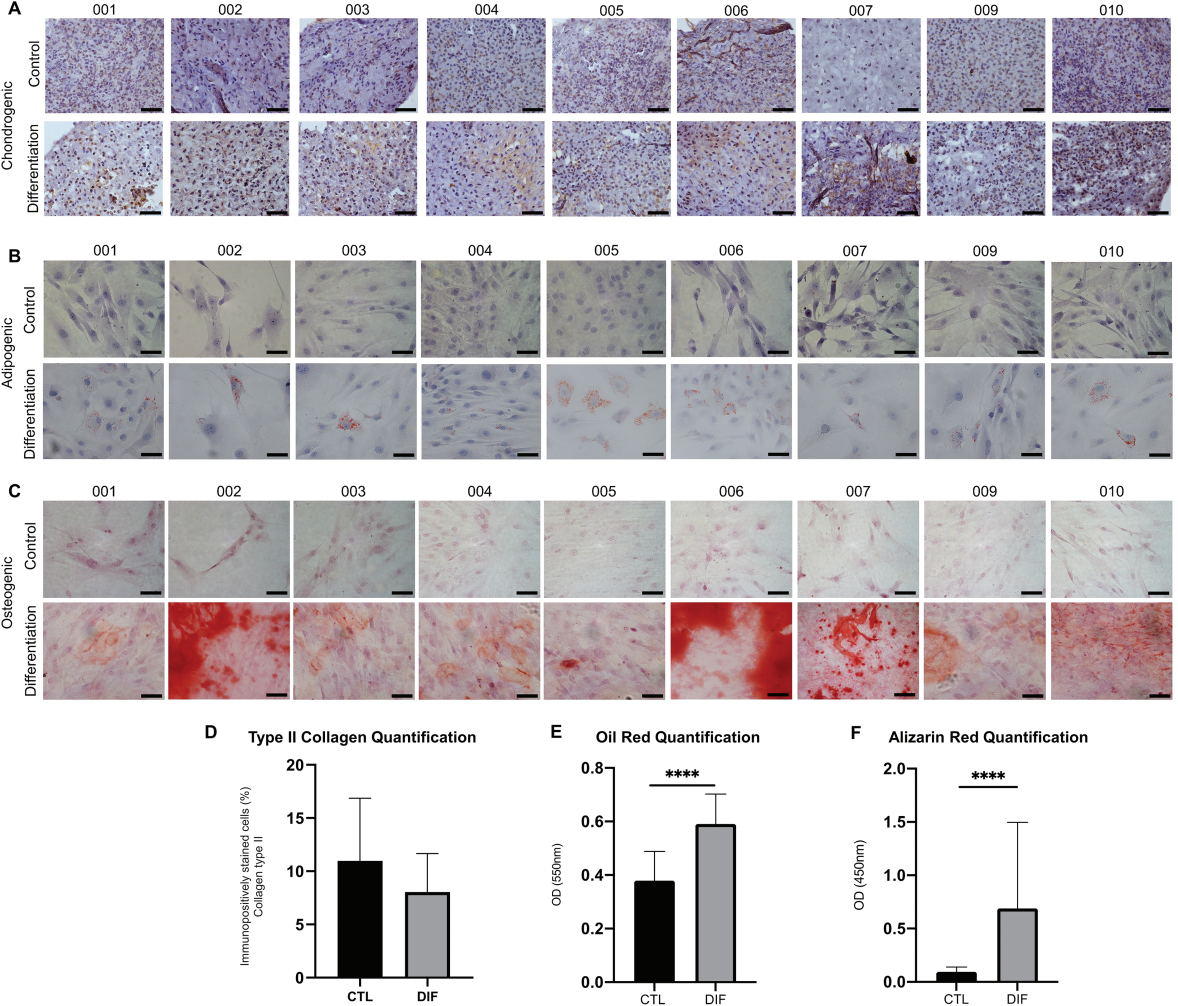
(A) In vitro chondrogenic differentiation. Cells were induced to chondrogenic differentiation (induced) or were not induced (control). Type II collagen immunohistological staining. (B) In vitro adipogenic differentiation. Cells were induced to adipogenic differentiation (induced) or were not induced (control). Oil Red O dye identifies the presence of lipid vacuoles inside the cells. (C) In vitro osteogenic differentiation. Cells were induced to osteogenic differentiation (induced) or were not induced (control). Brightfield and corresponding Alizarin Red stained identify calcium deposits produced by differentiated cells. (D) Percentage of type II collagen immunopositive cells. No significant difference between the control group and the cells induced to differentiation was observed. (E) Oil Red O quantification and (F) Alizarin Red S quantification. After differentiation, samples were submitted to the Oil Red O dye absorbance quantification assay to quantify the adipogenic differentiation and the Alizarin Red S dye absorbance quantification assay to quantify osteogenic differentiation. A statistical difference was observed for adipogenic and osteogenic differentiation (*P* < .01). Scale bar= 200 μm (A) and 50 μm (B and C).

The samples exhibited expression of type II collagen both prior to and subsequent to the initiation of chondrogenic differentiation, demonstrating their commitment to the chondrogenic lineage, as shown in Figure 1A. The quantitative evaluation of collagen type II immunopositive cells revealed no statistically significant distinction between the groups subjected to chondrogenic induction and the control (Figure 1F). Although nasoseptal chondroprogenitor cells were committed to the chondrogenic lineage, they were able to differentiate into adipocytes or osteoblasts when induced into these lineages (Figure 1B and Figure 1C). Cytoplasmic lipid droplets were accumulated in vitro in cultures of some human nasoseptal chondrogenic progenitor cells (Figure 1B). Similarly, nasoseptal chondrogenic progenitor cells could deposit considerable extracellular calcium under an osteogenic-inducing medium (Figure 1C). For adipogenic and osteogenic differentiation, the absorbance was quantified (Figure 1D and Figure 1E), and a statistically significant difference (*P* < .01) between cells induced to adipogenic or osteogenic differentiation and the not induced ones showed the ability of chondroprogenitor cells to respond to the specific induction.

**Figure 2.**
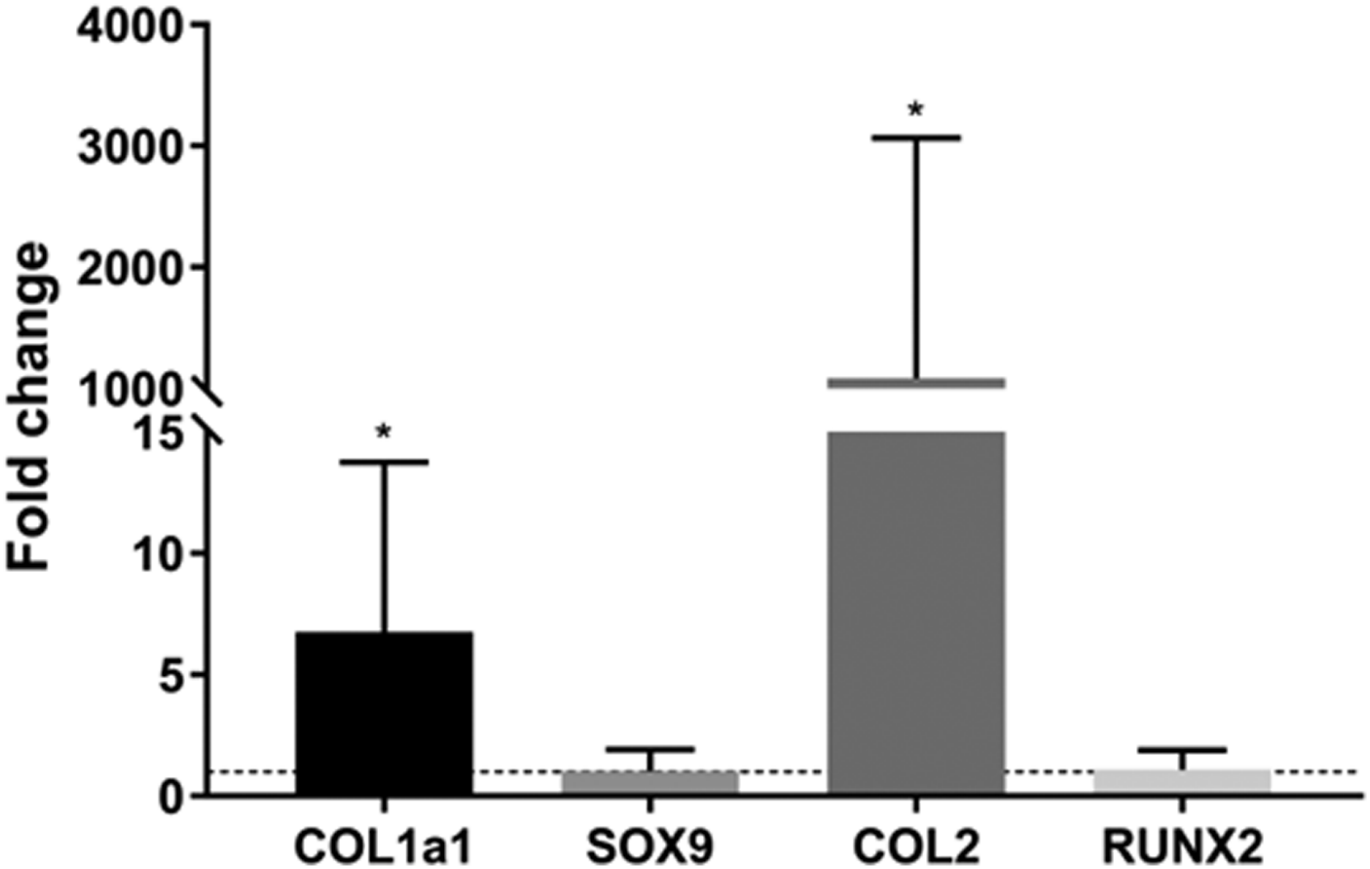
Relative expression analysis of genes related to chondrogenesis and osteogenesis after chondrogenic differentiation. The dotted line represents the expression at control (non-induced) condition. Statistical analysis was performed using the Mann-Whitney test. *P < .05.

The gene expression profile of nasoseptal chondroprogenitors (qPCR) showed that they increased their type I and type II collagen expression when differentiating in vitro into the chondrogenic lineage (Figure 2). The analysis of SOX9 expression showed that there is no difference between the induced and non-induced conditions after 21 days of cell culture (*P* < .05; Figure 2). This may indicate that the profile of their transcription factors may have already been defined in the analyzed cell culture passage and differentiation time. The same profile was observed for RUNX2, with no statistically significant difference to the control (*P* < .05), indicating that the analyzed cells could not reach a hypertrophic phenotype at this stage (Figure 2). Also, it is interesting to note that there is some variation in the gene expression between different cell donors (Supplementary Figure S2).

The main initial clinical complaints were severe or moderate TMJ arthralgia, TMJ crepitus, limited pain-free mouth opening, occlusal instability (5 out of 9 participants), crepitus (6 of 9 participants), and mandibular function limitation (5 out of 9 participants) 12 months after cell therapy injections, participants presented a decrease of arthralgia levels, improvement in mandibular function, increased maximum pain-free mouth opening, and absence of the TMJ crepitus (Supplementary Table S2).

### Medical complications during the clinical trial

In our clinical study, adverse events were systematically classified into 3 categories: non-serious, moderate, and severe, aligning with international clinical research guidelines and CNS Resolution no. 466, of October 12, 2012, Brazil.

We did not observe any severe adverse event related to autologous chondroprogenitor cells injection. A total of 23 adverse events were observed: 14 mild ones (mild TMJ pain, vertigo, masticatory muscle pain, night sweats, hyperhidrosis, purulent secretion on teeth), and 9 moderate ones (TMJ pain, headache, and gastritis) (Supplementary Table S3). The most frequent adverse reactions were TMJ arthralgia, masticatory muscular pain, and headache. Arthralgia and myalgia must probably be related to the injection procedure itself. It is noticeable that in TMJ pain has been improved over the follow-up period in all the research participants.

All events were recorded on a specific form. Our medical and dental team monitored each one until their resolution. Subsequently, the treatments administered were analyzed for their effectiveness in resolving health problems. All adverse events were promptly reported to the Research Ethics Committee (CEP) and submitted to the National Research Ethics Commission (CONEP). The events underwent a thorough evaluation by CONEP, and the clinical study continued as no serious adverse events were reported.”

### Autologous chondroprogenitor cells therapy reduced arthralgia

We used 2 pain assessment methods from the DC/TMD: (1) characteristic pain intensity and (2) chronic pain severity (Von Korff Scale).^27^

Autologous cell therapy reduced the pain intensity reported by all participants, with clinical improvement in the characteristic pain intensity over time (Table 2). All the participants reported pain improvement, and most of them considered that pain was absent, just 1 month after the experimental treatment. At the 6-month clinical follow-up, 78% of participants reported no pain. At 12 months after cell transplantation, 7 of the participants (78%) considered the pain absent, 2 (22%) considered the intensity as low (< 50), and no one as high (≥ 50).

**Table 2.** Clinical evaluation of joint pain during clinical follow-up.

Participant ID	Characteristic pain intensity					Chronic pain severity (Von Korff scale)				
	Initial	1 M after ACT	3 M after ACT	6 M after ACT	12 M after ACT	Initial	1 M after ACT	3 M after ACT	6 M after ACT	12 M after ACT
1	1	0	0	0	0	I	0	0	0	0
2	2	0	1	1	1	II	0	I	I	I
3	2	1	1	0	0	II	0	I	0	0
4	1	0	0	0	0	I	0	0	0	0
5	2	0	0	0	0	II	0	0	0	0
6	2	1	0	0	0	IV	II	0	0	0
7	0	0	0	0	0	0	0	0	0	0
8	1	—	—	—	—	1	—	—	—	—
9	2	*—*	2	2	1	I	—	I	II	I
10	2	—	—	0	0	I	—	—	I	I

(—) No clinical evaluation. Assessment of characteristic pain intensity, considered as: absent (0), low intensity (< 50) (1), or high intensity (≥ 50) (2). Clinical assessment of chronic pain severity, using the Von Korff scale, which classifies pain as absent (0), grade I, grade II, grade III, and grade IV.

The chronic pain severity analysis using the Von Korff Scale, also showed a reduction in the degree of pain-related disability after treatment. Before the chondroprogenitor cells injection, most participants considered the pain as high intensity (grade II) or with severe related disability (grade IV). One month after cell transplantation, most of participants considered joint pain absent (*n* = 6). At 12 months after cell injection, no research participant reported pain considered as grade II (high intensity), III or IV (with related disability) (Table 2).

### Autologous chondroprogenitor cells therapy promoted the stabilization of mandibular function

We observed that the therapy using autologous progenitor cells promoted the stabilization of the mandibular function. Among the 9 participants included, 4 had previous mandibular dysfunction (one intense, 2 moderate, and one mild degree; Supplementary Table S4). One month after the autologous cell therapy, the participant ID 5, with the worst and most severe mandibular dysfunction, completely improved and never presented it again. The 2 patients (ID 1 and 2) with moderate dysfunction also improved. The increase in the maximum range of pain-free mouth opening and maximum assisted mouth opening (Table 3), in addition to the stability of the clinical status in most of the participants, are evidence that the treatment prevented the progression of the TMJ degenerative disease, despite the overload produced by the orthognathic surgery procedure.

**Table 3.** Clinical analysis of pain-free maximum mouth opening and maximum assisted mouth opening.

Participant ID	Pain-free maximum mouth opening amplitude					Maximum-assisted mouth opening capacity				
	Initial	1 M after ACT	3 M after ACT	6 M after ACT	12 M after ACT	Initial	1 M after ACT	3 M after ACT	6 M after ACT	12 M after ACT
1	19*	26	30	31	35	23***	27	31	32	36
2	20*	33	37	37	39	50****	38	38	39	41
3	48**	40	47	55	58	55****	42	50	58	60
4	44**	42	47	48	46	51****	44	49	51	50
5	38**	35	37	39	42	48****	37	39	40	43
6	35**	38	45	45	45	39***	42	47	47	47
7	56**	45	46	48	50	56****	54	49	49	50
8	50**	—	—	—	—	54****	—	—	—	—
9	18*	—	26	32	30	20***	—	27	33	32
10	19*	26	30	31	35	29***	—	—	44	56

First columns correspond to the evaluation of pain-free maximum mouth opening amplitude: *values below ideal (<35 mm), and **above ideal (≥35 mm); second columns correspond to the maximum assisted mouth opening capacity: *** below ideal mean (< 40 mm); ****above ideal mean (≥40 mm).

There was a clinical improvement in almost all participants (88%, *n* = 8) concerning maximum pain-free mouth opening when comparing before and 12 months after treatment. Half of the participants were below the cutoff point (≥ 35 mm) at the time of inclusion in the clinical study and reached an opening range above this point after treatment. In the first month after cell injection, some participants showed a decrease in pain-free mouth opening amplitude. However, this impairment was possibly related to the orthognathic surgery and not to the cell injection procedure. A comparative analysis between the clinical segments showed that, from the first month onwards, all participants showed an improvement in the maximum pain-free mouth opening compared to the previous clinical segment (Table 3). Similar results were observed in the maximum assisted mouth opening analysis. 4 research participants below the expected average (≥ 40 mm) had a progressive improvement until the end of the follow-up period (Table 3).

### Autologous chondroprogenitor cells promoted the stability of condylar volume

Overall, only one participant showed loss of condylar volume on the right side (Participant ID 6), while 6 showed gain (Participant ID 1, 2, 5, 7, 9, 10) and 2 showed stable condyle (Participant ID 3 and 4). On the left side, 7 participants showed stability of condylar volume (Participant ID 1, 2, 3, 4, 7, 9); while 3, volume loss (Participant ID 5, 6, 10) (Supplementary Table S5).

### Autologous chondroprogenitor cells promoted joint tissue regeneration

Analyzing the initial and final CT scans (acquired 8 to 15 months after the therapy injection) (Table 4) and volumetric evaluation of the mandibular condyles before and after ACT (Supplementary Table S5), we could observe the following phenomena: recorticalization without volume change; completed regeneration, evidenced by recorticalization with volume increase; regeneration in progress, in which there is volume gain but without final recorticalization; stabilization, where cortical and volume are maintained; adaptive remodeling, with loss of volume leading to the recovery of joint space without discontinuous cortical in this specific region and therapeutic failure with discontinuous cortical structure and loss of volume. Although these phenomena can simultaneously occur in different areas of the same condyle, there is generally a predominance of one of them in the joint as a whole.

**Table 4.** Analyses of TMJ by CT exam before and after (8 to 15 months) ACT injection.

ID	Right		Left	
	Before	After	Before	After
01	Discontinuous cortical; Subchondral cysts; Decreased posterior joint space	Continuous cortical; Normal bone marrow density; Preserved joint spaces	Discontinuous cortical; Flattening; Hypodense bone marrow; Increased joint spaces	Continuous cortical; Anterior osteophytes; Hypodense bone marrow; Preserved joint spaces
02	Discontinuous cortical; Increased joint spaces;	Discontinuous cortical; Increased posterior joint space.	Discontinuous cortical; Increased joint spaces.	Discontinuous cortical; Subchondral cysts; Increased joint spaces.
03	Discontinuous cortical; Hypodense bone marrow; Preserved joint spaces	Discontinuous cortical; Hypodense bone marrow; Decreased posterior joint space	Discontinuous cortical; Hypodense bone marrow; Decreased joint spaces	Discontinuous cortical; Hypodense bone marrow; Decreased posterior joint space
04	Continuous cortical; Normal bone marrow density; Decreased joint spaces.	Continuous cortical; Normal bone marrow density; Decreased joint spaces.	Continuous cortical; Subchondral cysts; Increased joint spaces.	Continuous cortical; Normal bone marrow; Increased joint spaces;
05	Continuous cortical; Hypodense cortical; Flattening; Anterior osteophytes; Hypodense bone marrow; Preserved joint spaces.	Continuous cortical; Flattening; Anterior osteophytes; Hypodense bone marrow; Preserved joint spaces.	Continuous cortical; Anterior osteophytes; Hypodense bone marrow; Preserved joint spaces.	Continuous cortical; Anterior osteophytes; Hypodense bone marrow; Preserved joint spaces.
06	Discontinuous cortical; Decreased posterior joint space.	Discontinuous cortical; Increased posterior joint space.	Discontinuous cortical; Decreased posterior joint space.	Discontinuous cortical; Increased posterior joint space.
07	Discontinuous cortical; Flattening; Hypodense bone marrow; Decreased joint spaces.	Continuous cortical; Anterior osteophytes; Hypodense bone marrow; Preserved joint spaces.	Discontinuous cortical; Increased joint spaces.	Continuous cortical; Preserved joint spaces.
08	Continuous cortical; Hypodense bone marrow; Decreased joint spaces.	—	Discontinuous cortical; Hypodense bone marrow; Decreased joint spaces	—
09	Discontinuous cortical; Preserved joint spaces.	Discontinuous cortical; Hypodense bone marrow; Preserved joint spaces.	Continuous cortical; Flattening; Preserved joint spaces.	Continuous cortical; Flattening; Preserved joint spaces.
10	Discontinuous cortical; Normal bone marrow density; Preserved joint spaces.	Continuous cortical; Normal bone marrow density; Preserved joint spaces.	Discontinuous cortical; Normal bone marrow density; Preserved joint spaces.	Continuous cortical; Normal bone marrow density; Preserved joint spaces.

Abbreviation: ID, Participant ID.

**Figure 3.**
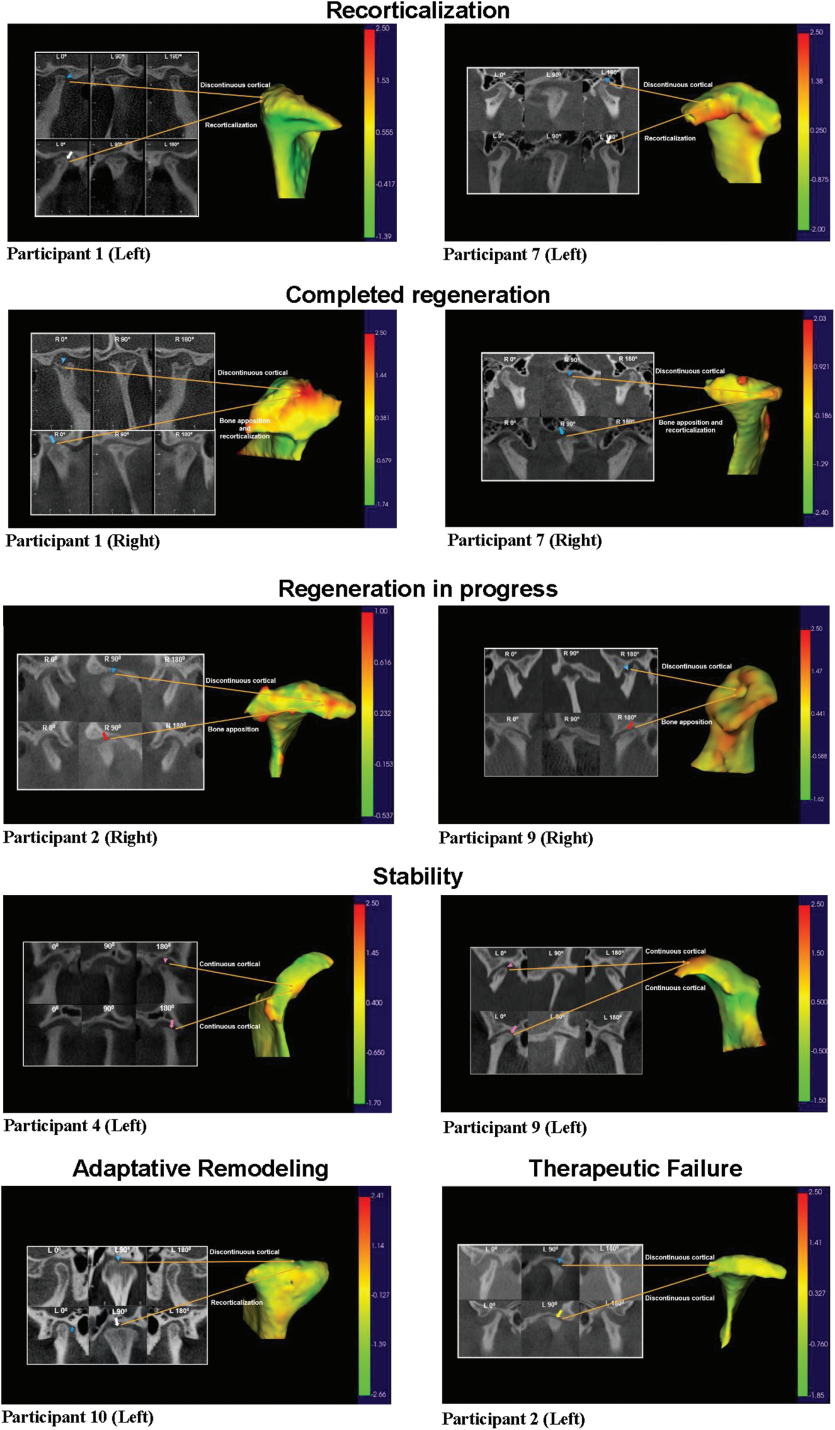
Image analysis by computed tomography of the TMJ joint and 3-dimensional remodeling images of condyles of participants before and after autologous chondroprogenitor cells joint injection. Right and left CT images from the patients before (upper line) and 8-15 months after (bottom line) the treatment: external lateral view (0^°^), frontal view (90^°^), and internal lateral view (180^°^). Prior to the initiation of the treatment, specific regions were demarcated using arrowheads, with the color blue designating instances of discontinuous cortical structure, and rose indicating continuous cortical structure. Subsequent to the treatment, the designated areas were redefined with arrows, wherein blue represented bone apposition and recorticalization, red signified bone apposition, white denoted recorticalization, rose represented continuous cortical structure, and yellow was assigned to discontinuous cortical structure. Additionally, the presence of a blue star was used to highlight areas undergoing adaptive remodeling. The color map indicates condylar resorption (negative value) and apposition (positive value) on the superimposed condylar surfaces (before and after treatment models). The orange arrows establish a correlation between the pre-treatment and post-treatment regions in the computed tomography (CT) scans and their respective counterparts observed in the 3-dimensional superimposed images.

Figure 3 shows images categorized based on the predominant phenomena observed in the selected condyles, with 2 condyles allocated to each group:

CT scans with the respective 3D reconstructions and superimposition of the pre-treatment and post-treatment models, showing the areas of apposition of bone tissue (positive values). The intensity of the orange or red color is directly related to the amount of new bone tissue formed (Figure 3). The intensity of yellow or green is directly related to tissue balance or resorption. Areas with a value next to zero indicate a balance between apposition and resorption (Figure 3). Tissue regeneration promoted by chondroprogenitor cells was shown by cortical continuity and new areas of bone tissue on CT images. In addition, in the 3-D reconstructions, the areas of bone regeneration are indicated by color intensity of positive values (Figure 3).

## Discussion

Most currently available conservative treatment strategies for CR focus only on symptom management^28,29^ with limited success and not addressing or halting disease progression^30^ Currently, TMJ CR cases do not respond to conservative or minimally invasive therapies, and frequently evolves to surgical intervention and prostheses implantation.^28^

However, ideally, TMJ CR treatment should aim not only at relieving pain and restoring function but also preventing the progression of cartilage and subchondral bone loss, targeting orthopedic stability. Biological cartilage repair techniques are emerging, with methods such as autologous transplantation of chondrocytes or chondroprogenitor cells showing encouraging results.^31^ Our group recently reported the first case of intra-articular injection of cultured autologous cells from nasal septum to control TMJ CR, with CT images evidencing articular bone regeneration.^20^

Researchers still deal with the challenge of chondrocyte expansion in vitro.^32^ Isolation and expansion of cartilage cells probably involve at least chondrocytes and their progenitors. The different obtention protocols for each cell type and their membrane markers after expansion are still controversial.^32^ The cells injected in this clinical trial were isolated following the protocol described by Amaral et al,^18^ which obtained chondroprogenitor cells from the nasal septum fibrocartilage perichondrium cambium layer with surface markers characteristic of mesenchymal progenitor cells.

Cells obtained from cartilage tissue after culture passages^33^ or in an inflammatory environment,^34^ were demonstrated to suffer dedifferentiation, starting to express clusters of differentiation (CD) characteristic of mesenchymal stem cells (MSC).^33^ They also start to synthesizing collagen I^34^ and displaying multi-potent stem/progenitor cell characteristics,^33^ such as anti-inflammatory and immune-modulatory activities. So, they could suppress the injury-induced production of inflammatory mediators within joint tissues, which are responsible for at least part of clinical symptoms.^35^ MSCs preferentially attracted to and harbored in diseased tissue rather than intact tissue. When intra-articular injected, they bind not only into cartilage but also bone defects, proliferate and participate in their regeneration.^36^

The expression of CD146 increases after subsequent culture passages, as verified as part of the dedifferentiation process in other studies.^33,37^ As described elsewhere^18^ by part of our research group, in the first passages of the nasal septum chondroprogenitors subculture (one to 3), cells were positive for surface markers described for mesenchymal stem cells, except CD146. The absence of this perivascular cell marker is consistent with their avascular niche in fibrocartilage. On the other hand, CD146 + cells can also recreate a hematopoiesis-supportive human bone-like tissue^26,38^ supported a developmental hierarchy of skeletal progenitors in humans where a purified population of PDPN+ CD146- CD73+ CD164+ cells serially generate colony forming units from single cells in vitro and multi-lineage ossicles containing bone, cartilage, and stroma upon sub-renal transplantation in mice.

Therefore, it is still unclear whether fibrocartilage stem cells (FCSC) are quiescent stem cells that are left from earlier development, remain reserved in tissue, and then become reactivated upon in vitro culture or, instead, whether most mature chondrocytes have such potential to become progenitors.

Most of the nasal septum cultured cells (≥ 90%) injected into 8 of the 9 research participants expressed CD146 membrane marker in the latter passages (3 to 5). As an exception, 49.2% of the analyzed cell population, at passage 4, of research participant ID 09, a 61-year-old woman, was negative for CD146. With increasing age, a decrease in the dedifferentiation capacity of MSC has already been verified,^39^ and also in the expression of the CD146 marker. This was probably reflected in the behavior of the isolated and injected cells of this specific research participant, which promoted the higher volume of tissue formation of the entire sample (45.33%). The possible combination of different cell types (chondroprogenitor cells and dedifferentiated chondrocytes) within the injury site can lead to a synergistic action between them, simultaneously forming cartilage and bone through different growth processes.

Furthermore, the cells isolated presented a statistically significant increase in type II and type I collagen expression in the group submitted to chondrogenic differentiation compared to the control group. Interestingly, the cells of participant ID 09, expressed high levels of SOX9 and type 2 collagen in control group compared to induced group (Supplementary Figure S2). This different expression pattern may be related to distinct cell populations that behave differently to the same stimulus.

All these characteristics and behaviors of nasal septum progenitor cells may control the progression of DJD and potentially promote the formation of new tissue. DJD are better classified through CT images, regardless of painful symptoms, being also fundamental in the follow-up of the disease’s natural history or response to treatment.^7^ Qualitative and quantitative volumetric analyzes were previously performed to assess the stability of severe TMJ OA in its natural course. The presence of a continuous cortex determined the absence of notable volumetric losses at a mean 2 years follow-up.^6^ Although most areas of cortical erosion (74.4%) converted into continuous cortex during this period, the mean volume loss was close to 15%. Mandibular condyle cortical line continuity and density were also related to condylar volume changes after orthognathic surgery. Considering a loss of condylar volume of less or equal than 10% as a sign of stability, the continuous cortex group showed a higher stability rate (92.5%) compared to the discontinuous cortex group (51.3%).^6^

In this clinical trial, analyzing 8-15 months CT images follow-up, it was possible to observe the formation of appositional tissue at the upper surface of the mandibular condyles, either with initially discontinuous cortical, hypodense but continuous cortical, or normal continuous cortical. The exceptions were condyles presenting continuous cortex but deformity in these regions. Analyzing the initial and final volume of the 6 condyles with a gain of volume, we observed a medium volume increase of 21.33% (Supplementary Table S5). Both bone apposition and resorption areas are considered when evaluating the volume, and even when condyle volume was considered stable, bone apposition was noticed. Apposition was observed in the superior and posterior surfaces, commonly subjected to bone resorption. It was possible to identify areas of apposition very close to or precisely in the same areas of previous cortical discontinuity (Figure 3). Our hypothesis for this new tissue formation is that the cortical discontinuity or its low density allowed the contact of the injected cells with the vascular niche of the bone marrow, which may have led the cartilage progenitor cells to differentiate into osteoblasts. Direct contact between FCSC and endothelial cells significantly enhanced their osteogenic differentiation.^40^ On the other hand, when the injected progenitor cells do not establish direct contact with the endothelial cells, they remain at the cartilage lesion’s surface, forming cartilaginous tissue that can later be replaced by bone tissue through a process resembling endochondral ossification.^41^ FCSC is also shown to be able to form transient cartilage and initially inhibit angiogenesis to generate an avascular cartilage. Later, pro-angiogenic growth factors may be released to promote the proliferation of endothelial cells and form bone tissue.^40^ Another possibility is direct transdifferentiation, where hypertrophic chondrocytes could directly differentiate into osteoblasts and form bone.^42^

The hypothesis that CR after orthognathic surgery is part of a progressive process of pre-surgical resorption was previously evaluated by 3D condylar remodeling. The condylar volume during the presurgical phase was considered relatively stable (−3.3 ± 37.2 mm^3^). However, condylar volume suffered a significant reduction during the post-surgical phase, on an average of −12.2%.^43^ In this clinical trial, 12 condyles decreased their volume after orthognathic surgery despite cell therapy injection. However almost all of them (8 of 12) presented a volume loss lower or equal than 10%, which was previously considered as stability of volume for such cases.^6^ The average percentage of loss observed in this clinical trial, considering 4 condyles, was 27.82% (Supplementary Table S5).

In terms of safety, the outcomes of the reported cases were consistent with previous studies using culture-expanded autologous cells and administered via intra-articular route the managing DJD in a various other pathologic conditions.^44^ The procedure was generally well tolerated with only a mild and brief increase in discomfort noted immediately after injecting the therapy. No cell-related serious adverse events or abnormalities in laboratory parameters or clinical signs were observed, potentially being a safe and valuable therapy if evidence accumulate.

The results suggest that autologous cartilage progenitors can become a suitable treatment for the control of TMJ arthralgia and related disability secondary to CR, with possible regenerative properties of joint tissues. This therapeutic approach should be considered in patients refractory to traditional conservative treatments before escalating to more invasive surgical interventions.

## Conclusion

The reported therapies proposed for the treatment of temporomandibular degenerative diseases show that a single intra-articular injection of autologous chondrocytes, expanded in vitro and expressing some of the markers of cartilage progenitors, was safe and well tolerated during the 12-month follow-up period. Using clinical and image analyses, we report improved arthralgia and mandibular function, and favorable structural improvements in TMJ tissues affected by condylar resorption. Further investigations should accumulate sufficient evidence to propose new strategies to treat temporomandibular degenerative diseases refractory to other therapies.

## Supplementary material

Supplementary material is available at *Stem Cells Translational Medicine* online.

szae026_suppl_Supplementary_Figures_and_Tables

## Data Availability

The data that support the findings of this study are available from the corresponding author upon reasonable request.
