# Supplementary material for: Nasal septum-derived chondroprogenitor cells control mandibular condylar resorption consequent to orthognathic surgery: a clinical trial
Source: Stem Cells Transl Med. 2024 Apr 12;13(7):593–605. doi: 10.1093/stcltm/szae026 (PMC11227969; doi:10.1093/stcltm/szae026)
Supplement: szae026_suppl_Supplementary_Figures_and_Tables [file szae026_suppl_supplementary_figures_and_tables.zip › Supplementary_Table_S5_220324f.docx]

**Supplementary Table S5**. Comparative volumetric evaluation of the

mandibular condyles before and after *autologous* *chondroprogenitor*

*cells therapy (ACT)* injection.

| **Participant ID** | **Right TMJ** | | | | **Left TMJ** | | | |
| --- | --- | --- | --- | --- | --- | --- | --- | --- |
|  | **Inicial** | **12 M after ACT** | **Difference before and after ACT** | **%** | **Inicial** | **12 M after ACT** | **Difference before**  **and after ACT** | **%** |
| *1* | 886.80 | 943,85 | +57,05 | **+6.43%** | 962,32 | 895,50 | -66.82 | **-6.94%*** |
| *2* | 725.20 | 797.0 | +71.8 | **+9.9%** | 699,9 | 644.0 | -55.9 | **-7.99%*** |
| *3* | 471.3 | 461.9 | -9.4 | **-1.99%*** | 558.0 | 527.5 | -30.5 | **-5.47%*** |
| *4* | 1186.0 | 1061.9 | -124.10 | **-10.46%*** | 316.1 | 308.6 | -7.5 | **-2.37%*** |
| *5* | 692.6 | 747.9 | +55.3 | **+7.98%** | 982.6 | 856.6 | -126.0 | **-12.82%** |
| *6* | 970.46 | 640.5 | -329.96 | **-34%** | 961.14 | 701,43 | -259.71 | **-27%** |
| *7* | 788.5 | 1054.2 | +265.7 | **+33.7%** | 1118.5 | 1059.1 | -59.4 | **-5.31%*** |
| *9* | 457,1 | 664.3 | +207.2 | **+45,33%** | 447.2 | 413.0 | -34.2 | **-7.65%*** |
| *10* | 2154.1 | 2686.4 | +532.32 | **+24.71%** | 1864.1 | 1165.4 | -698.7 | **-37.48%** |

Condylar bone volume before and after ACT at the right and left side. *Initial and final volumetric difference (%)

considered stable. A stable condyle was defined as a loss of condylar volume less than or equal to 10% (Sun et al., 2021).
