# Supplementary material for: Nasal septum-derived chondroprogenitor cells control mandibular condylar resorption consequent to orthognathic surgery: a clinical trial
Source: Stem Cells Transl Med. 2024 Apr 12;13(7):593–605. doi: 10.1093/stcltm/szae026 (PMC11227969; doi:10.1093/stcltm/szae026)
Supplement: szae026_suppl_Supplementary_Figures_and_Tables [file szae026_suppl_supplementary_figures_and_tables.zip › Supplementary_Table_S1_220324.docx]

**Supplementary Table S1**. Primers sequence for qPCR analysis.

| Official Symbol | Sequência (5’->3’) | Anneling temperature |
| --- | --- | --- |
| *COL1A1* | Fw: AGGGCTCCAACGAGATCGAGATCCG  Rv: TACAGGAAGCAGACAGGGCCAACGTCG | 60°C |
| *COL2A1* | Fw: CATCCCACCCTCTCACAGTT  Rv: GCCTCTGCCTTGACCCGAAG | 60°C |
| *SOX9* | Fw: AAGAACAAGCCGCACGTCAA  Rv: CCGTTCTTCACCGACTTCCTC | 60°C |
| *RUNX2* | Fw: ACTGGCGCTGCAACAAGAC  Rv: CCCGCCATGACAGTAACCA | 55°C |
| *POLR2A* | Fw: TACCACGTCATCTCCTTTGATGGCT Rv: GTGCGGCTGCTTCCATAA | 60°C |

*COL1A1*: collagen type I alpha 1 chain, *COL2A1*: collagen type II alpha 1 chain, *SOX9*: SRY-box transcription factor 9, *RUNX2*: RUNX family transcription factor 2, *POL2RA*: RNA polymerase II subunit A (POLR2A). Fw: Forward primer, Rv: Reverse primer.
