# Supplementary material for: Nasal septum-derived chondroprogenitor cells control mandibular condylar resorption consequent to orthognathic surgery: a clinical trial
Source: Stem Cells Transl Med. 2024 Apr 12;13(7):593–605. doi: 10.1093/stcltm/szae026 (PMC11227969; doi:10.1093/stcltm/szae026)
Supplement: szae026_suppl_Supplementary_Figures_and_Tables [file szae026_suppl_supplementary_figures_and_tables.zip › Supplementary_Table_S2_220324ff.docx]

**Supplementary Table S2**. Clinical status of participants, at the moment of inclusion and at the end of the clinical follow-up, 12 months after *autologous* *chondroprogenitor cells therapy (ACT)* injection.

| **Participant ID** | **Age** | **Gender** | **Inicial Clinical Status** | **Final clinical status (12 months after cell transplantation)** |
| --- | --- | --- | --- | --- |
| 001 | 27 | M | Decreased maximum pain-free mouth opening;  TMJ crepitation (right and left);  Mandibular functional limitation. | Increased maximum mouth opening;  No TMJ crepitus (right and left);  Mandibular function improvement. |
| 002 | 24 | F | Moderate TMJ arthralgia;  Decreased maximum pain-free mouth opening;  TMJ crepitus (right);  Mandibular functional limitation. | Mild TMJ arthralgia;  Increased maximum pain-free mouth opening;  TMJ crepitus (right);  Mandibular functional limitation. |
| 003 | 22 | F | Moderate TMJ arthralgia;  TMJ joint crepitus (right and left);  Occlusal instability. | No TMJ arthralgia;  TMJ joint crepitus (right and left);  Absence of occlusal instability. |
| 004 | 20 | F | Mild TMJ arthralgia;  TMJ crepitus (right and left);  Occlusal instability. | No TMJ arthralgia;  No TMJ crepitus;  Occlusal instability. |
| 005 | 47 | F | Moderate TMJ arthralgia;  TMJ crepitus (right and left);  Mandibular functional limitation;  Mandibular locking;  Occlusal instability. | No TMJ arthralgia;  TMJ joint crepitus (right and left);  Mandibular function improvement;  No mandibular locking;  Absence of occlusal instability. |
| 006 | 29 | M | Severe TMJ arthralgia;  Decreased maximum pain-free mouth opening  TMJ crepitus (right and left);  Occlusal instability. | No TMJ arthralgia;  Increased maximum pain-free mouth opening;  TMJ crepitus (right);  Occlusal instability. |
| 007 | 18 | M | TMJ crepitus (right and left). | TMJ crepitus (left). |
| 008 | 25 | F | Moderate TMJ arthralgia;  Mandibular functional limitation; | - |
| 009 | 61 | F | Moderate TMJ arthralgia;  Decreased maximum pain-free mouth opening;  Mandibular functional limitation;  Occlusal instability. | Mild TMJ arthralgia;  increased maximum pain-free mouth opening;  Mandibular functional limitation;  Improved occlusal instability. |
| 010 | 33 | F | Moderate TMJ arthralgia;  Mandibular functional limitation;  Decreased maximum pain-free mouth opening. | No TMJ arthralgia;  Mandibular functional limitation;  Increased pain-free maximum mouth opening. |
