# Supplementary material for: Nasal septum-derived chondroprogenitor cells control mandibular condylar resorption consequent to orthognathic surgery: a clinical trial
Source: Stem Cells Transl Med. 2024 Apr 12;13(7):593–605. doi: 10.1093/stcltm/szae026 (PMC11227969; doi:10.1093/stcltm/szae026)
Supplement: szae026_suppl_Supplementary_Figures_and_Tables [file szae026_suppl_supplementary_figures_and_tables.zip › Supplementary_Table_S3_220324f.docx]

**Supplementary Table S3**. Clinical complications presented by research participants over 12 months after *autologous* *chondroprogenitor cells therapy (ACT)* injection.

| **Classification of Adverse Event** | | **Number of participants who had the adverse event** | **Frequence of the adverse event** |
| --- | --- | --- | --- |
| Mild adverse event | Mild TMJ pain | 5 | 7 |
|  | Vertigo | 1 | 1 |
|  | Nausea | 0 | 0 |
|  | Fainting | 0 | 0 |
|  | bruise | 0 | 0 |
|  | Lameness | 0 | 0 |
|  | Joint effusion | 0 | 0 |
|  | Masticatory Muscle Pain | 2 | 4 |
|  | Night sweats | 1 | 1 |
|  | hyperhidrosis after morning exercise | 1 | 1 |
|  | Purulent secretion on teeth | 1 | 0 |
|  | **TOTAL** |  | **14** |
| Moderate adverse event | Moderate TMJ pain | 3 | 3 |
|  | Headache | 4 | 4 |
|  | Fever | 0 | 0 |
|  | TM trauma | 0 | 0 |
|  | Pseudarthrosis | 1 | 1 |
|  | gastritis | 1 | 1 |
|  | **TOTAL** |  | **9** |
| Severe adverse event | Severe TMJ pain | 0 | 0 |
|  | Infecction | 0 | 0 |
|  | Vascular damage | 0 | 0 |
|  | Nerve damage | 0 | 0 |
|  | Death | 0 | 0 |
|  | **TOTAL** |  | 0 |
