# Supplementary material for: Nasal septum-derived chondroprogenitor cells control mandibular condylar resorption consequent to orthognathic surgery: a clinical trial
Source: Stem Cells Transl Med. 2024 Apr 12;13(7):593–605. doi: 10.1093/stcltm/szae026 (PMC11227969; doi:10.1093/stcltm/szae026)
Supplement: szae026_suppl_Supplementary_Figures_and_Tables [file szae026_suppl_supplementary_figures_and_tables.zip › Supplementary_Table_S4_220324.docx]

**Supplementary Table S4**. Mandibular function limitation analyses of participants.

| ***Participant ID*** | **Mandibular Function Limitation** | | | | |
| --- | --- | --- | --- | --- | --- |
|  | **Inicial** | **1 M after ACT** | **3 M after ACT** | **6 M after ACT** | **12 M after ACT** |
| *1* | 2 | 2 | 1 | 1 | 1 |
| *2* | 2 | 1 | 2 | 2 | 1 |
| *3* | 0 | 0 | 1 | 0 | 0 |
| *4* | 0 | 1 | 1 | 0 | 0 |
| *5* | 3 | 0 | 0 | 0 | 0 |
| *6* | 0 | 0 | 0 | 0 | 0 |
| *7* | 0 | 0 | 0 | 0 | 0 |
| *8* | 2 | - | - | - | - |
| *9* | 1 | - | 2 | 3 | 2 |
| *10* | 0 | - | - | 1 | 1 |

Assessment of mandibular functional limitation, considered as: absent (0), mild (1), moderate (2),

intense (3) and severe (4).
